# Deploying Clinical Decision Support for Familial Hypercholesterolemia

**DOI:** 10.1055/s-0040-1721489

**Published:** 2020-12-31

**Authors:** Hana Bangash, Joseph Sutton, Justin H. Gundelach, Laurie Pencille, Ahmed Makkawy, Omar Elsekaily, Ozan Dikilitas, Ali Mir, Robert Freimuth, Pedro J. Caraballo, Iftikhar J. Kullo

**Affiliations:** 1 Department of Cardiovascular Medicine, Mayo Clinic, Rochester, Minnesota, United States; 2 Department of Information Technology, Mayo Clinic, Rochester, Minnesota, United States; 3 Center for Science of HealthCare Delivery, Mayo Clinic, Rochester, Minnesota, United States; 4 User Experience Research, Saharafox Creative Agency, Rochester, Minnesota, United States; 5 Department of Digital Health Sciences, Mayo Clinic, Rochester, Minnesota, United States; 6 Department of General Internal Medicine, Mayo Clinic, Rochester, Minnesota, United States

**Keywords:** implementation and deployment, facilitators and barriers, testing and evaluation, clinical decision support, precision medicine, workflows and human interactions, human–computer interaction, interfaces and usability

## Abstract

**Objective:**

Familial hypercholesterolemia (FH), a prevalent genomic disorder that increases risk of coronary heart disease, remains significantly underdiagnosed. Clinical decision support (CDS) tools have the potential to increase FH detection. We describe our experience in the development and implementation of a genomic CDS for FH at a large academic medical center.

**Methods:**

CDS development and implementation were conducted in four phases: (1) development and validation of an algorithm to identify “possible FH”; (2) obtaining approvals from institutional committees to develop the CDS; (3) development of the initial prototype; and (4) use of an implementation science framework to evaluate the CDS.

**Results:**

The timeline for this work was approximately 4 years; algorithm development and validation occurred from August 2018 to February 2020. During this 4-year period, we engaged with 15 stakeholder groups to build and integrate the CDS, including health care providers who gave feedback at each stage of development. During CDS implementation six main challenges were identified: (1) need for multiple institutional committee approvals; (2) need to align the CDS with institutional knowledge resources; (3) need to adapt the CDS to differing workflows; (4) lack of institutional guidelines for CDS implementation; (5) transition to a new institutional electronic health record (EHR) system; and (6) limitations of the EHR related to genomic medicine.

**Conclusion:**

We identified multiple challenges in different domains while developing CDS for FH and integrating it with the EHR. The lessons learned herein may be helpful in streamlining the development and deployment of CDS to facilitate genomic medicine implementation.

## Background and Significance

Tier 1 genomic disorders including familial hypercholesterolemia (FH), Lynch syndrome, and hereditary breast and ovarian cancer syndromes affect approximately 1 in 100 individuals in the United States.^1^ FH, the most prevalent of these conditions, continues to have poor awareness, detection, and control.^2,3^ The uptake of cascade testing for FH is also low and approximately 90% of individuals with FH in the United States remain undiagnosed.^4,5^ The current generation of electronic health record (EHR) systems is not well equipped for genomic medicine implementation; instead of increasing provider efficiency and simplifying clinical workflows, EHRs add to provider cognitive burden.^6,7^ Genomic medicine implementation through the development and use of digital tools including clinical decision support (CDS), has the potential to provide evidence-based treatment recommendations, knowledge resources, and actionable order sets to health care providers at the point-of-care; facilitating FH awareness, improving early detection and treatment, and promoting cascade testing of at-risk family members.^8^ In this report, we provide an overview of the process, timeline, and challenges encountered in the development and implementation of a CDS tool for FH, a relatively common genomic disorder with substantial public health implications, at a large academic institution.

## Methods

Our aim was to develop a CDS tool for FH based on provider feedback that could be implemented in varying clinical workflows including primary care and specialist clinics at Mayo Clinic. The process of CDS development and implementation occurred in four phases.

### Development and Validation of a Phenotyping Algorithm to Trigger CDS

An electronic phenotyping algorithm to identify “possible FH” (low-density lipoprotein cholesterol [LDL-C] ≥ 190 mg/dL, in the absence of any secondary causes of hypercholesterolemia) was developed using expert input to inform modifications to the previously validated and published “Screening Employees And Residents in the Community for Hypercholesterolemia (SEARCH)” algorithm.^9^ The modified algorithm subsequently underwent validation by two physicians, who manually reviewed a subset of 50 patient EHRs; 25 in whom the alert fired and 25 in whom the alert did not fire.

### Clinical Approval

To initiate CDS development, we first had to submit a request to the institutional CDS subcommittee within the Electronic Health Record/Revenue Cycle Management committee. An “EHR Change Request Form” was required to detail our intended goals and was presented to the CDS subcommittee for review. Upon approval from the CDS subcommittee, we had to obtain additional approval from leadership in the department of cardiovascular medicine. This step required us to review existing institutional knowledge resources on FH such as “AskMayoExpert” which contains a dedicated topic module on FH. We aligned our CDS content with AskMayoExpert and other institutional knowledge resources to ensure consistency across all tools and resources. Additionally we worked with the Mayo Clinic Knowledge Management and Delivery unit, information technology (IT), and the Center for the Science of Healthcare Delivery to align the CDS with an institutional knowledge platform called “MayoExpertAdvisor” that also provides recommendations on FH. This process required transferring the triggering algorithm and CDS content to the MayoExpertAdvisor team so that they could align their recommendations with the CDS.

### Initial Prototype and Modifications

An initial CDS prototype was created based on feedback from physician focus groups and a knowledge elicitation survey as previously described.^10^ Input from the focus groups and survey informed the structure and content of the initial prototype. This initial prototype underwent two prototyping iterations– the first iteration was informed by a heuristic evaluation in which Nielsen’s 10 heuristic principles were applied.^11^ Two user experience designers and one human factors designer conducted the heuristic evaluation and provided their expert recommendations on how to improve the CDS tool. The second CDS iteration was informed by feedback from the Mayo Clinic CDS subcommittee that recommended aligning the tool with existing institutional Best Practice Advisory (BPA) style guidelines. The BPA style guidelines provided specifications for optimizing the design of CDS tools. The prototyping iterations resulted in two CDS prototype formats: a passive alert (BPA) and an asynchronous message (in-basket). An IT project lead from the “portal and decision support” unit was then assigned to begin building the CDS tool within the EHR.

### An Implementation Science Framework to Evaluate CDS

**Fig. 1:**
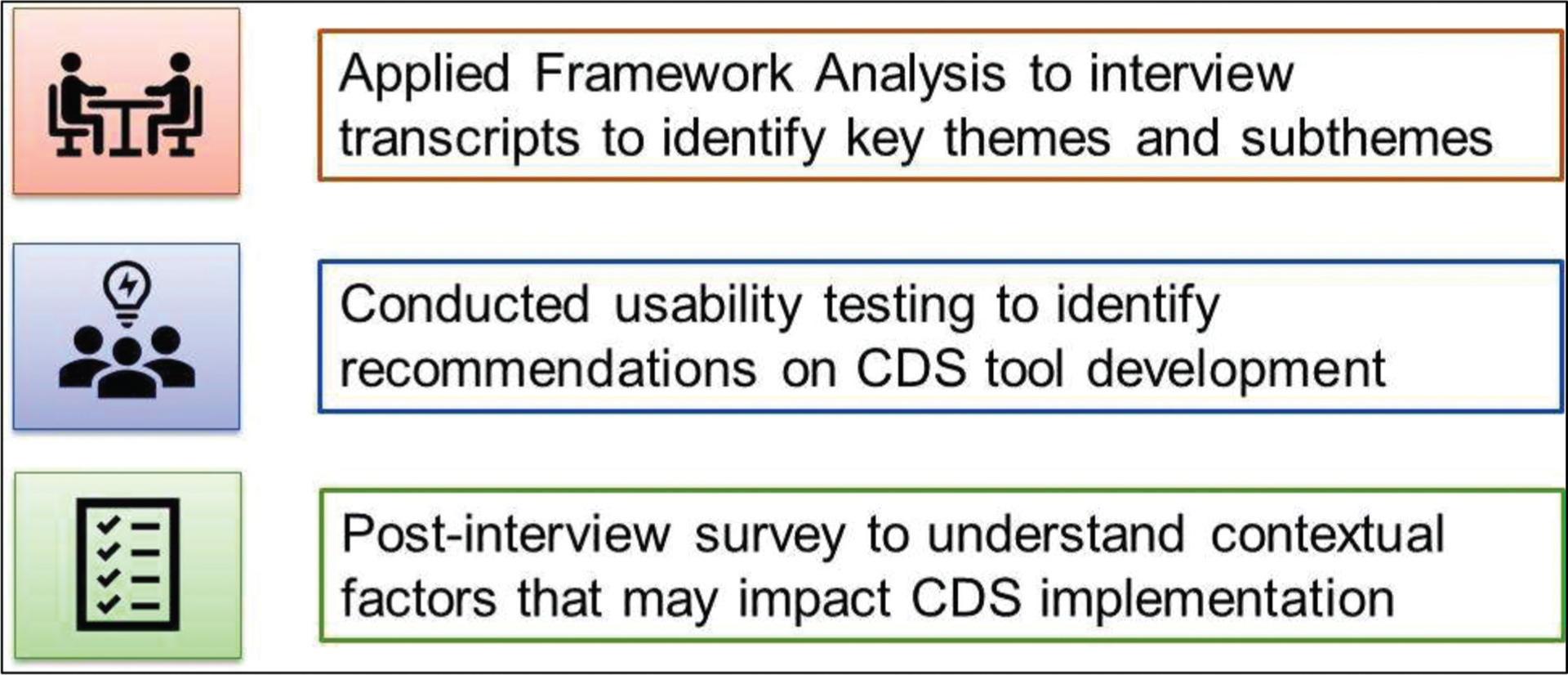
An implementation science framework was used to gain provider perspectives on the clinical decision support (CDS) tool for familial hypercholesterolemia (FH) through qualitative interviews, usability testing, and a post-interview implementation survey to assess variables likely to impact CDS uptake in clinical practice.

To further refine the CDS formats, a second qualitative study was conducted to understand provider perspectives on the CDS tool and to identify varying clinical workflows across the institution for better CDS integration. An implementation science framework was used to conduct qualitative provider interviews and usability testing and a post-interview implementation survey was completed by providers to identify variables that could influence CDS adoption in clinical practice (Fig. 1). Provider input resulted in rapid prototyping and iterative refinements to the FH CDS.

## Results

**Fig. 2:**
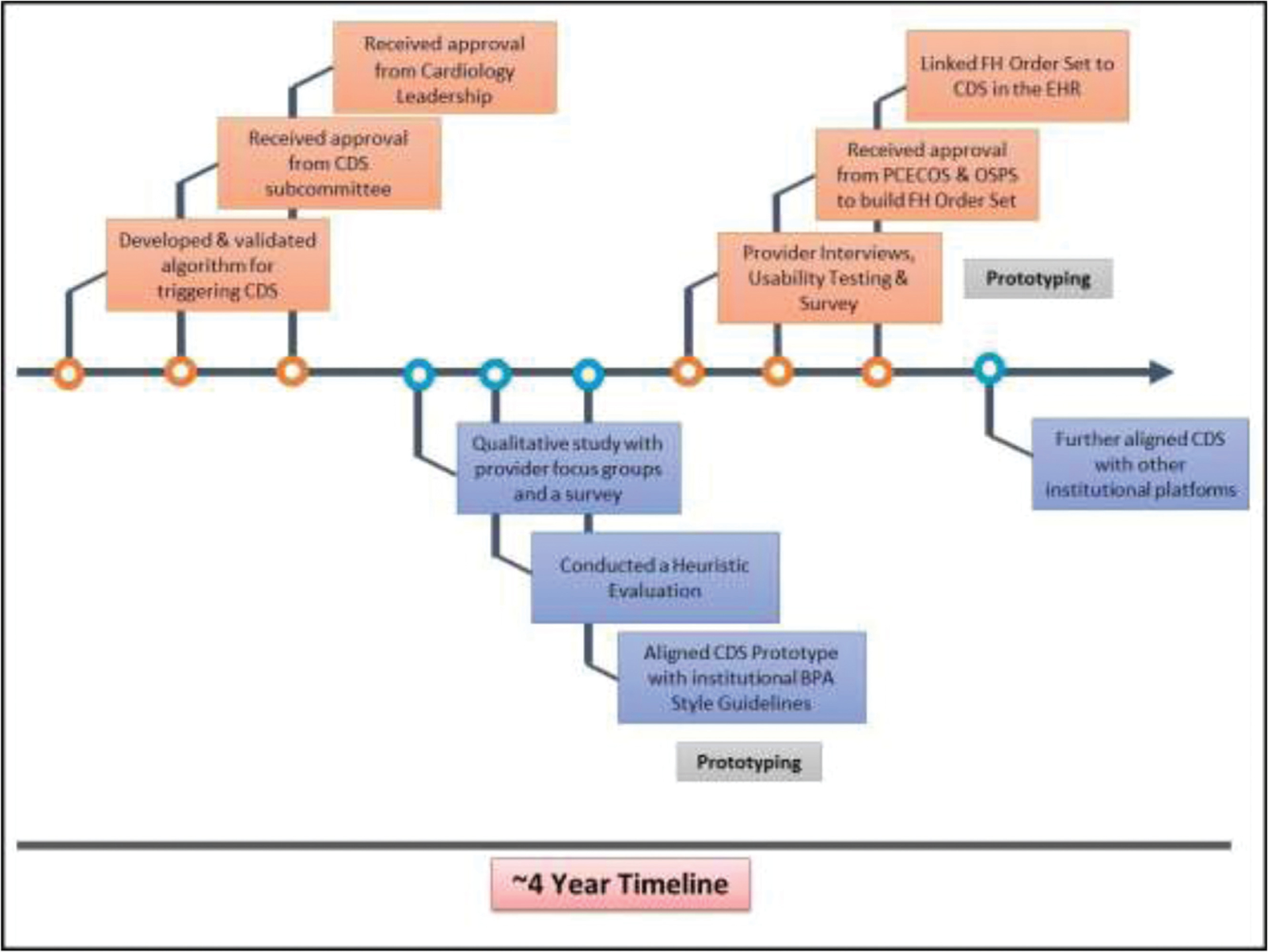
The process of clinical decision support (CDS) development and electronic health record (EHR) integration occurred over 4 years and included algorithm development, multiple institutional committee approvals, qualitative research to obtain provider feedback on the CDS, as well as multiple rounds of prototyping and iterative refinements.

**Fig. 3:**
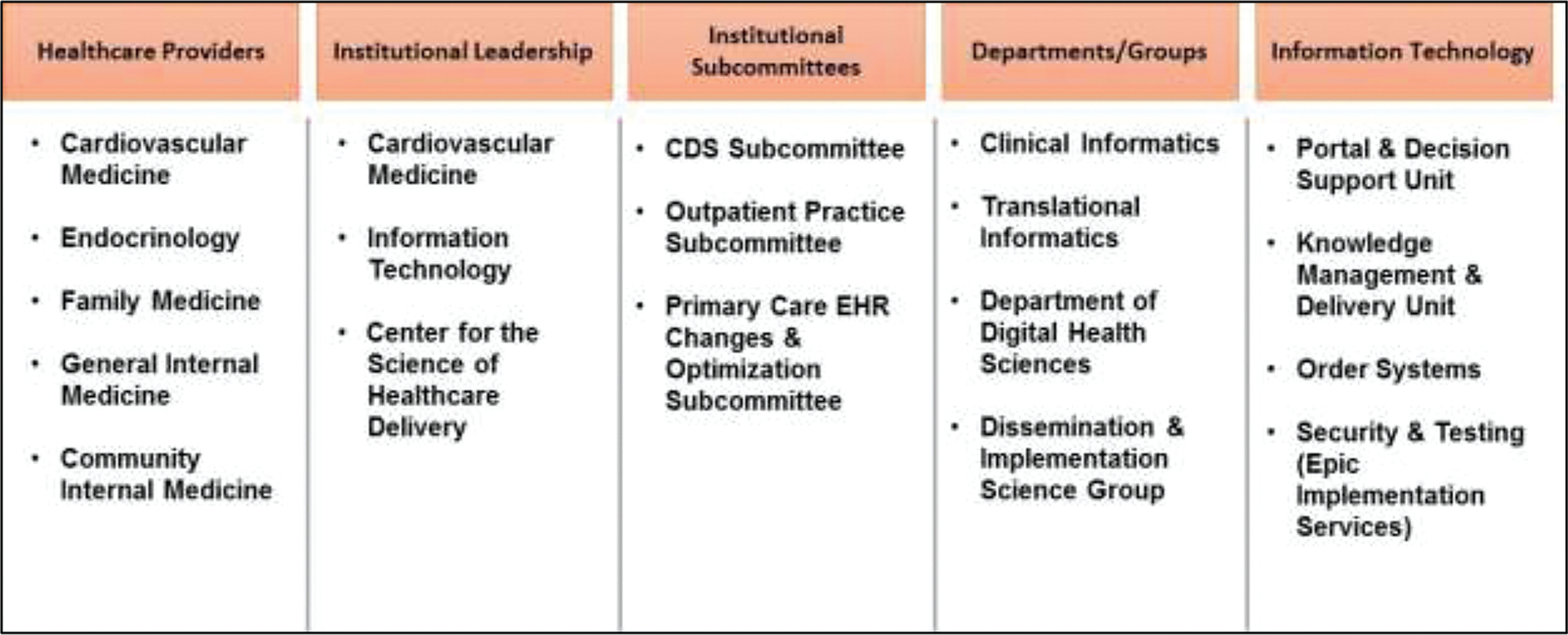
During clinical decision support (CDS) development, we engaged with 15 different stakeholder groups to build and implement the CDS tool for familial hypercholesterolemia (FH). Health care providers from five different departments participated in each stage of CDS development.

The total time taken for CDS development and EHR integration was approximately 4 years with algorithm development and validation taking approximately 18 months (Fig. 2). During this 4-year period, we engaged with 15 different stakeholder groups, including health care providers who gave input at each stage of CDS development and evaluation; institutional leadership and subcommittees provided the approvals necessary for initiation of CDS development; and several departments and groups across the institution along with various units within IT participated in CDS development and final deployment (Fig. 3).

CDS algorithm validation results revealed that of the 25 individuals who triggered the alert, all met algorithm criteria and were appropriately identified as “possible FH.” Of the 25 individuals with LDL-C ≥ 190 mg/dL in whom the alert did not fire, all were appropriately excluded based on the defined exclusion criteria: (1) age < 18 years or > 80 years; (2) secondary causes of hypercholesterolemia documented within the past 365 days including elevated thyroid-stimulating hormone > 10 mIU/L, elevated alkaline phosphatase > 200 IU/L, elevated urine protein > 3 g/24 hours; (3) an existing FH diagnosis code; or (4) prior visit to the institutional FH clinic. In this limited patient subset, the CDS triggering algorithm performed with 100% accuracy in detecting individuals with “possible FH.” Upon completion of validation, the algorithm was integrated with the institutional EHR and linked to trigger the CDS alert at the point-of-care. Data collected through algorithm deployment will be used to iteratively refine the algorithm criteria and conduct additional validation through EHR review.

Qualitative studies with providers highlighted important themes to consider during the development process, while the implementation science framework identified key contextual variables, at the level of the institution and the provider, which could influence clinical adoption of the tool. The multiple rounds of rapid prototyping and iterative refinements ensured that the CDS was well-suited for implementation in differing workflows across the institution.

In both qualitative studies conducted during the CDS development process, a key finding was the need for a dedicated FH order set linked to the CDS. Providers indicated that an easily accessible FH order set embedded within the CDS tool could significantly reduce the number of clicks needed to place orders, streamline clinical workflows, and increase efficiency. This feedback led to the development of an FH-specific order set titled “Familial Hypercholesterolemia Smart Set.” To build the FH order set we submitted an “EHR Change Request Form” that was reviewed by the Primary Care EHR Changes and Optimization Sub-committee (PCECOS). The PCECOS review process took approximately 3 months, and upon approval, the order set request was moved forward to the Order Set/Protocol Sub-committee (OSPS) for a second approval, which was received within a week. The department of Nursing/Clinical Informatics assigned a project lead to build a proof-of-concept FH order set using an existing institutional order set for hypercholesterolemia as a template. The template order set was modified to include appropriate diagnosis options, medications, laboratory tests, and a referral to the institutional FH clinic. Certain laboratory tests as well as the referral to the institutional FH clinic were autopopulated within the order set based on provider feedback. The FH order set was then linked to the CDS and integrated with the EHR.

## Discussion

In this report we describe the process, timeline, and challenges encountered during implementation of CDS for a genomic disorder, at a large academic institution. Several barriers were encountered during the 4-year period of CDS development and implementation. First, the need for multiple institutional committee approvals for both the CDS and FH order set resulted in significant delays. Second, the need to align the CDS content with existing institutional knowledge resources and platforms was also time intensive. Third, differing workflows identified in the primary care setting as compared with the specialist clinics necessitated that the CDS tool and triggering algorithm be modified multiple times. Fourth, the lack of institutional guidelines for genomic CDS implementation meant that many of the initial steps taken during the development process were based on trial and error, resulting in a nonlinear process with time delays. Fifth, in 2018 Mayo Clinic transitioned to a new EHR system which affected the IT department’s capacity to work on this project. Lastly, limitations of the EHR resulted in some provider feedback not being implemented in the final CDS tool.

Though genomic CDS has the potential to promote disease awareness and facilitate disease detection and treatment at the point-of-care, there is a need to scale the process of CDS implementation in the current generation of EHRs.^12^ Possible strategies to scale genomic CDS implementation include: (1) streamlining institutional processes to expedite deployment of knowledge delivery and CDS tools; (2) incorporating provider feedback in building and integrating CDS tools at each stage of development; (3) obtaining insights into multilevel contextual factors relevant to CDS implementation early on in the process of development; (4) addressing and removing institutional barriers to implementation; (5) developing CDS implementation guidelines that can be standardized across institutions; and (6) considering an “App”-based model for digital health tools whereby crowdsourcing can be used to build genomic CDS apps that can then be layered onto a standardized, interoperable EHR.^13^

Our next steps in CDS implementation include an enterprise wide go-live of the CDS tools followed by a study to assess outcomes post-deployment, in particular provider satisfaction with the alert. We are also working on generating computable representations of genomic data that can trigger CDS, to further our efforts in genomic medicine implementation.

## Conclusion

We identified multiple challenges in different domains while deploying a genomic CDS for FH at an academic medical center. These challenges were nontrivial and need to be addressed at institutional and national levels. The lessons learned herein can be used to streamline CDS development and deployment to facilitate genomic medicine implementation.
